# Pathogenicity and Pathological Characteristics of African Swine Fever Virus Strains from Pig Farms in South Korea from 2022 to January 2023

**DOI:** 10.3390/pathogens12091158

**Published:** 2023-09-13

**Authors:** Ki-Hyun Cho, Seong-Keun Hong, Da-Young Kim, Min-Kyung Jang, Jong-Ho Kim, Hyunkyoung Lee, Eun-Mi Kim, Ji-Hoon Park, Tae-Young Suh, Jun-Gu Choi, Dae-Sung Yoo, Hae-Eun Kang, Yeon-Hee Kim

**Affiliations:** 1 Foreign Animal Disease Division, Animal and Plant Quarantine Agency, Gimcheon 39660, Republic of Korea; vet10@korea.kr (K.-H.C.); hongsky@korea.kr (S.-K.H.); kdy04207@naver.com (D.-Y.K.); mkjang0506@korea.kr (M.-K.J.); jhpark93@korea.kr (J.-H.P.); sty8911@korea.kr (T.-Y.S.); happythomas@korea.kr (J.-G.C.); kanghe@korea.kr (H.-E.K.); 2 Animal Disease Diagnostic Division, Animal and Plant Quarantine Agency, Gimcheon 39660, Republic of Korea; whdgh2339@korea.kr (J.-H.K.); ieustina@korea.kr (H.L.); kimem256@korea.kr (E.-M.K.); 3 College of Veterinary Medicine, Chonnam National University, Gwangju 61186, Republic of Korea; shanuar@jnu.ac.kr

**Keywords:** African swine fever virus, pig farm, South Korea, pathogenicity, pathology

## Abstract

Since the first African swine fever (ASF) outbreak occurred at a pig farm in South Korea in September 2019, as of 31 January 2023, 31 ASF cases have occurred at pig farms, while 2799 ASF virus (ASFV)-infected wild boars have been identified. The circulation of ASFV in wild boar populations poses a high risk of spillover to pig farms in the country. However, information on the changes in the pathogenicity of Korean ASFV strains from wild boars is not available. Investigating the pathogenicity of ASFV strains from pig farms is the only way to predict their alterations. In a previous study, no changes in the pathogenicity of ASFV strains circulating during 2019–2021 were identified through animal experiments. In this study, we chose two ASFV strains with potentially reduced pathogenicity among ten viruses obtained from pig premises from 2022 to January 2023 and estimated their pathogenicities and pathological characteristics. All the inoculated pigs died 8–10 days post–inoculation after showing pyrexia, depression, anorexia, and recumbency together with the common pathological lesions of enlarged hemorrhagic lymph nodes and splenomegaly with infarction. These results support that the pathogenicity among ASFV isolates in South Korea still remained unchanged during the study period.

## 1. Introduction

African swine fever (ASF) is a contagious disease that affects only swine species. The characteristic symptoms of ASF include acute hemorrhagic fever and subsequent death. The highly virulent ASF virus (ASV) can induce up to 100% lethality. The economic impact of the disease is significant owing to direct losses attributed to high mortality rates and trade restrictions. Although ASF is endemic to certain African countries, it has now spread to other continents, such as Asia, Europe, America, and Oceania, reaching a pandemic phase [1]. ASF can be transmitted via direct contact between infected and susceptible pigs and contaminated fomites, such as vehicles, equipment, and clothes [2]. Multiple vaccine candidates demonstrated promise in terms of commercialization; however, establishing their efficacy and safety is still challenging [3]. Since no effective treatment is currently available for ASFV, control measures based on strict biosecurity and stamping-out policies are considered the only feasible methods for disease control [4].

ASFV, which is a linear double-stranded DNA virus, is the only member of the *Asfarviridae* family and *Asfivirus* genus. The ASFV genome ranging from 170 to 190 kbp in length encodes more than 150 proteins [5]. ASFV strains are divided into 24 genotypes based on the partial sequence of the *B646L* gene encoding the p72 protein [6]. ASFV can be further intra-genotyped by analyzing multiple genomic sites. The genes commonly used for sub-genotyping are the *EP402R* gene encoding the CD2v protein (serogroups 1–8), the *E183L* gene encoding the p54 protein (genotypes I–XXIV), central variable region within *B602L* (CVR1, CVR1/SNP1–3, and CVR2), and the intergenic region (IGR) between *I73R* and *I329L* (IGR I–IV) [7,8,9,10]. Recently, various gene markers, such as *O174L*, *K145R*, multigene family (MGF) *505*, and IGRs between *MGF 505 9R* and *10R* have been employed to distinguish ASFV strains within the same genotype [10,11]. Genetic analysis of select genes can be useful for elucidating the source of the virus and transmission patterns, thereby contributing to the effective control of ASF.

The phenotypes of ASFV isolates have traditionally been classified into the following three main groups according to virulence: highly, moderately, and low virulent isolates. Four clinical courses have been classified in pigs: peracute, acute, subacute, and chronic forms. In the peracute form, caused by the highly virulent ASFV, the disease is quickly fatal with a short duration and reaches 100% mortality. This form is characterized by sudden death with few signs. The acute form is the most common clinical presentation of a natural infection, which is induced by highly virulent ASFV strains. Clinical signs, including fever, loss of appetite, apathy, recumbency, skin erythema, breathing difficulties, nasal hemorrhage, and diarrhea were observed in animal experiments. Infected pigs succumbed to death in 6–20 days, with a mortality rate of nearly 100%. The subacute form of ASF, caused by moderately virulent ASFVs, shows clinical signs similar to the acute form; however, it is generally milder and lasts longer. Infected pigs usually die 15–45 days post-inoculation (dpi). The mortality rates range from 30% to 70%. The chronic form is caused by low virulent isolates with a clinical course of 2–15 months. The affected pigs may succumbed to death after the infection, but the mortality rate is low [2]. The pathogenicity of the ASFV strain can be indirectly estimated by considering the clinical signs and gross lesions at necropsy at the farm of origin and changes in the viral genome. A challenge experiment with pigs can be used to evaluate pathogenicity directly.

In South Korea, fourteen cases of ASF were reported on pig farms in 2019, followed by two and five outbreaks at pig farms in 2020 and 2021, respectively [12,13,14]. Ten ASFV-infected pig farms were confirmed between 2022 and January 2023. Meanwhile, 2799 ASFV-infected wild boars had been identified in 33 cities/counties from October 2019 to January 2023. ASFV has been circulating in wild boar populations in the country for over three years, posing a possibility of spillover to pig farms in the country. ASFV strains with reduced pathogenicity have been reported in certain Baltic countries following extensive epidemics in wild boars over the past few years [15,16,17]. Low virulent genotypes I and II ASFV isolates were identified between 2020 and 2021 in China [18,19]. These imply the possibility of attenuated viruses being introduced into both pig farms and wild boar populations in South Korea. However, little information is available on the genetic characteristics and pathogenicity of ASFV strains isolated from wild boars in South Korea.

A previous study suggested that the pathogenicity of ASFV isolates circulating in South Korea during 2019–2021 could be highly virulent based on animal experiments using four selected strains isolated from pig farms [20]. In this study, we chose two ASFV strains that had potentially reduced pathogenicity among the ASFV isolates from pig farms and conducted a challenge experiment to estimate the changes in the pathogenicity and pathogenic characteristics of ASFV strains circulating in South Korea from 2022 to January 2023.

## 2. Materials and Methods

### 2.1. Virus

Among the ten ASFV-positive pig farms confirmed from 2022 to January 2023, seven farms were detected by farmers’ notifications, and the other three (4th, 8th, and 9th) were found by active surveillance. At the six notified farms, except for the 5th farm, the observed clinical signs were inappetence, fever, abortion, and death. At the 5th pig farm, only inappetence and abortions were observed. The major gross lesions observed in the pig farms with deceased pigs were infarction and enlargement of the spleen, enlargement and hemorrhage in the lymph nodes, and petechiae in the kidneys and liver. For the three pig farms detected by active surveillance in the quarantine zone, slaughterhouse, and another farm owned by owners of the infected farm, no clinical signs were observed (Table 1).

For the animal experiment, we chose two ASFV strains with potentially reduced pathogenicity, considering the disease outbreak situation, clinical signs and gross lesions observed at pig farms, diagnostic results, epidemiological associations, and genetic analysis. The first strain, Korea/Pig/Hongcheon/2022, was isolated from the ASFV-infected pig farm reported in Hongcheon County on 26 May 2022. This outbreak had the widest time interval since the last outbreak in October 2021. The second strain, Korea/Pig/Pocheon/2023, was isolated from the 8th pig farm occurred on 5 January 2023. The outbreak was detected through active surveillance at a slaughterhouse. Infected pigs at the abattoir did not show any clinical signs, and their diagnosis results revealed that five of six ASFV-positive pigs had 33–38 cycle threshold (Ct) values by WOAH TaqMan^®^ quantitative polymerase chain reaction (qPCR). No clinical manifestations were observed in the other pigs at the presumed origin. ASFV strains isolated from three pig farms (4th, 5th, and 9th) were excluded due to the epidemiological associations with other ASFV-infected farms and diagnostic results presenting with 15–20 Ct values of most ASFV-positive pigs. All ten strains belonged to p72 genotype II and CD2v serogroup 8 with central variable region (CVR) 1 variant. One strain from 10th pig farm was of intergenic region between I73R and I329L (IGR) I variants, while the other nine strains were of IGR II [21]. At the whole genome level, ASFV strains from 1st–7th and 10th pig farms had 99.97–99.99% homology compared with the first Korean ASFV strain (Korea/Pig/Paju1/2019), which was highly pathogenic [20], while two isolates from 8th and 9th pig premises were 99.66% identical to Korea/Pig/Paju1/2019. In particular, 620 bp of deletion was detected in MGF 110–5L-6L and MGF110–7L in Left Variable Region of the 8th and 9th ASFV strains. The genome sequence homology between 1st and 8th ASFV strains was 99.64%. The two ASFV strains were isolated from the spleens of dead pigs from affected pig premises. The virus was isolated from the samples according to the procedure of the Center for Animal Health Research, European Union Reference Laboratory of ASF and as described previously [20]. 

### 2.2. Experimental Design

Twelve 8-week-old landrace pigs were purchased from a commercial farm. The farm of origin, which is located in Uiseong County, Gyeongsangbuk Province, is a farrow-to-finish farm with high biosecurity and hygiene standards, which was free from important swine infectious diseases, including ASF, classical swine fever, porcine respiratory and reproductive syndrome, porcine epidemic diarrhea, porcine parvovirus, and foot-and-mouth disease. The disease status and vaccine program were as previously described [20]. All the pigs were negative for ASFV antigens and antibodies using WOAH TaqMan^®^ qPCR and the Eurofins Ingenasa^®^-INGEZIM PPA COMPAC K3 kit (Eurofins Ingenasa, Madrid, Spain). Animal experiments were conducted under Animal Biosafety Level 3 (ABSL-3) of the Animal and Plant Quarantine Agency (APQA) and approved by the Animal Ethics Committee of APQA (authorization no. 2023–709, approved on 16 January 2023). In the ABSL-3, temperature was maintained at 23.0 ± 2.0 °C with 50.0 ± 20.0% of humidity during the pig experiment. The minimum density per pig of less than 25 kg was 0.54 m^2^. Pigs were provided with ropes for chewing as environmental enrichment. All the pigs were introduced into ABSL-3 and randomly divided into three groups. All the animals were allowed five days of acclimatization considering conditions of minimizing the risk of heat stress in pigs by transportation. To assess the pathogenicity of the two isolates, two groups of five pigs were intramuscularly (IM) injected with 10^3^ HAD_50_ of their respective strains. Groups 1 and 2 were inoculated with Korea/Pig/Hongcheon/2022 and Korea/Pig/Pocheon/2023 strains, respectively. Group 3 consisted of two pigs and was a control group (mock-infected group).

### 2.3. Clinical Presentation

All the pigs in Groups 1–3 were examined daily from the day of inoculation (0 dpi) until death. The clinical signs in each pig were measured according to previously described ASF guidelines [22]. The clinical scoring criteria were divided into ten categories with score ranges, namely rectal temperature (0–5), inappetence (0–6), recumbency (0–6), skin changes (0–3), joint swelling (0–4), respiratory difficulties with coughing (0–3), ocular discharge (0–1), diarrhea/bloody diarrhea (0–4), hematuria (0–4), and vomiting (0–4). The maximum score was 40 points. A humane endpoint was determined according to that which was previously described [17]. The pigs subjected to euthanasia were sedated, anesthetized, and exsanguinated as in the previous study [20]. Dead pigs were immediately subjected to necropsy. Pigs in Group 3 were euthanized in the same way when all the pigs in Groups 1 and 2 died.

### 2.4. Sample Collection

Whole blood treated with EDTA, serum, and swab samples from the oral and nasal cavities and rectum were obtained from each pig from 0 dpi to the day before death as in the previous study [20]. All the samples except for serum were used to assess the ASFV load using WOAH TaqMan^®^ qPCR [23]. The serum samples were screened for the presence of antibodies against ASFV. All the pigs were subjected to necropsy immediately following death or euthanasia. Samples from tissues, namely the spleen, liver, kidneys, heart, forelimb muscles, and submaxillary, gastrohepatic, gastric, renal, and mesenteric lymph nodes, were collected. ASFV loads in the collected tissue samples were also assessed.

### 2.5. Viral Load Analysis and Detection of Anti-ASFV Antibody

Whole blood, swab, and tissue samples were analyzed for ASFV nucleic acids using extraction of viral nucleic acids and WOAH TaqMan^®^ qPCR and the viral genome load was estimated as in the previous study [20]. ASFV antibody detection in the serum samples was performed using a commercial enzyme-linked immunosorbent assay (ELISA) kit (Eurofins Ingenasa^®^-INGEZIM PPA COMPAC K3 kit; Eurofins Ingenasa, Madrid, Spain), according to the manufacturer’s instructions. If ELISA results indicated that samples were positive or inconclusive, the immunoperoxidase test was performed according to the standard protocols provided by the European Reference Laboratory for ASF.

### 2.6. Histopathology

Samples from tissues, namely the spleen, liver, kidneys, heart, lung, small intestines, large intestines, and submandibular, tracheobronchial, gastrohepatic, renal, and mesenteric lymph nodes, which were collected from all pigs in the experiment, were fixed in 10% neutral buffered formalin. Representative sections of each tissue were cut, embedded in paraffin, and sectioned at 4 µm. After hematoxylin and eosin staining of sectioned paraffin-embedded tissues, histopathological changes were classified into four categories: normal (0), mild (1), moderate (2), and severe (3), according to the ASF pathology guideline [24].

### 2.7. Statistical Analysis

Bayes factor was used to analyze differences between two groups for statistical significance. Statistical analyses were conducted in R 4.3.1 (https://r-project.org accessed on 24 July 2023). 

## 3. Results

### 3.1. Disease Progression and Observed Clinical Signs

In Group 1, fever (>40 °C) was first detected at 5.4 ± 0.5 (5–6) dpi followed by the observation of clinical signs at 6.6 ± 0.9 (5–7) dpi (Table 2). The fever continued until death in all five pigs, excluding pig no. 1, in which the rectal temperature decreased to normal (39.3 °C) the day before its death. The clinical signs commonly observed in Group 1 were fever, depression, anorexia, recumbency, labored breathing, and ocular discharge. Some pigs exhibited joint swelling, ocular discharge, diarrhea, melena, and epistaxis (Table 3). The maximum clinical score immediately before death ranged from 8 to 24 points. All the pigs in this group died at 9.8 ± 0.4 (9–10) dpi (Table 2).

**Figure 1:**
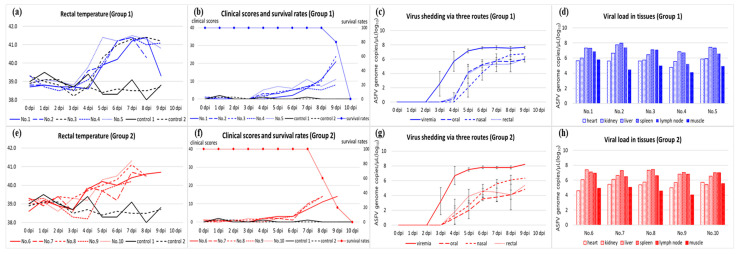
Results of rectal temperature (**a**,**e**), survival rate and clinical score (**b**,**f**), viremia and virus shedding via oral, nasal, and rectal routes (**c**,**g**), and viral load in heart, kidneys, liver, spleen, submandibular lymph node, and forelimb muscles (**d**,**h**) of Groups 1 and 2.

**Figure 2:**
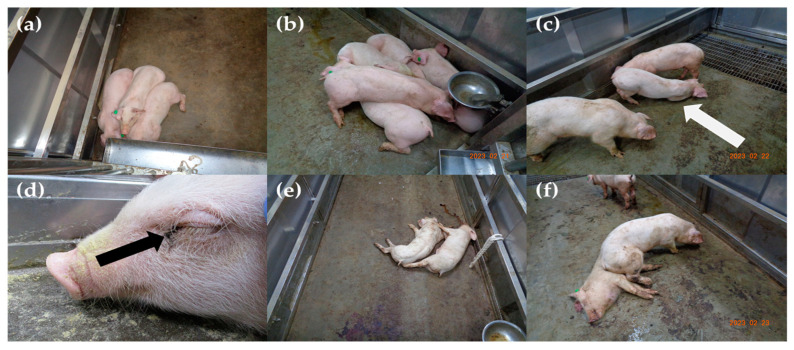
Clinical signs of pigs infected with two Korean ASFV strains (Korea/Pig/Hongcheon/2022 and Korea/Pig/Pocheon/2023) isolated from 2022 to January 2023. (**a**,**b**) huddled together in Group 1 at 5 dpi and Group 2 at 7 dpi, respectively, (**c**) astasia due to the severe joint swelling in the right forelimb (white arrow) of pig no. 7 at 8 dpi, (**d**) ocular discharge (black arrow) of pig no. 5 at 9 dpi, and (**e**,**f**) melena and death in Group 1 at 10 dpi and in Group 2 at 8 dpi, respectively.

The disease dynamics in Group 2 were similar to those in Group 1. In Group 2, rectal temperature increased to more than 40 °C at 5.6 ± 0.9 (5–7) dpi. Clinical manifestations were observed after 7.0 days. All the pigs presented with fever, depression, inappetence, and recumbency. Joint swelling, diarrhea, and bloody diarrhea were observed in some pigs. The clinical scores of all the pigs, excluding pig no. 9, continued to increase until 10 points before death. The survival period of this group was 8.8 ± 0.8 (8–10) dpi. The control group did not exhibit any clinical signs. The rectal temperature between the control group and Group 1 and between the control group and Group 2 during the experimental period was different with substantial evidence (BF10 = 7.45, 3.36). There was no difference in the mean total survival days and clinical scores between Groups 1 and 2. The clinical characteristics and signs of each pig are summarized in Table 2 and Table 3. The evolution of the rectal temperature, clinical score, and survival rate is shown in Figure 1a,b,e,f. Clinical signs that were observed in Groups 1 and 2 are shown in Figure 2.

### 3.2. Viral Load in Blood, Virus Shedding via Oral, Nasal, and Rectal Routes, and Antibody Detection

In Group 1, the ASFV genome was first detected in the blood at 3.2 ± 0.4 (3–4) dpi, which started 2–3 days earlier than the onset of pyrexia. On the first day of detection in the blood, viral loads were 10^2^–10^4^ copies/µL and increased to more than 10^7^ copies/µL two days later. A high viral load was maintained in all the pigs until their death. Pigs in Group 1 began to excrete ASFV via oral and nasal routes at 4.6 ± 0.5 (4–5) and 5.2 ± 0.4 (5–6) dpi, respectively, which was 1–2 days after the beginning of viremia. A viral load of 10^0^–10^4^ copies/µL in both routes was observed on the first day of detection and increased to more than 10^5^ copies/µL. Viral excretion via the rectal route started at 4.8 ± 0.4 (4–5) dpi. At the beginning of detection, the viral load in the rectal swab was 10^1^–10^5^ copies/µL. Its maximum level was 10^5^–10^6^ copies/µL. The ASFV genome in the blood and three swab samples was detected from the first detection to the death of the pigs. Viral shedding via the three routes in Group 1 is shown as the average ± standard deviation in Figure 1c. Antibodies against ASFV were not detected in any serum from Group 1.

Group 2 demonstrated a similar pattern of viremia and virus shedding via the oral, nasal, and rectal routes. The detection of the viral genome began at 3.2 ± 0.4 (3–4) dpi. Viral load in the blood increased from 10^3^–10^4^ copies/µL to more than 10^7^ copies/µL in two days after the beginning of viremia. This high viral titer continued until death. Virus shedding via the oral and nasal routes started at a level of less than 10^2^–10^3^ copies/µL at 4.8 ± 0.8 (4–6) and 4.4 ± 0.5 (4–5) dpi, respectively. Virus excretion via both routes reached a maximum viral load of 10^3^–10^6^ copies/µL within 1–3 days after their first detection. Rectal shedding began at 4.4 ± 0.5 (4–5) dpi and its viral load increased from 10^2^–10^4^ copies/µL to 10^4^–10^5^ copies/µL. Virus shedding via the three routes in Group 2 is shown as the average ± standard deviation in Figure 1g. All sera from Group 2 were negative for the antibody against ASFV.

### 3.3. Viral Load in the Tissues and Post-Mortem Lesions

Seven organs, namely the spleen, submandibular lymph nodes, liver, kidneys, heart, lung, and forelimb muscles, of all the pigs were subjected to WOAH TaqMan^®^ qPCR. On average, the viral load in the spleen was the highest at 10^7.22^ copies/µL, followed by the liver (10^7.05^ copies/µL) and lymph node (10^6.70^ copies/µL). The mean viral load of the kidneys and heart was 10^5.89^ and 10^5.36^ copies/µL, respectively. The forelimb muscles had the lowest viral titers at 10^4.84^ copies/µL on average. The distribution of the virus in the tissues and individual pigs varied slightly (Figure 1d,h).

**Figure 3:**
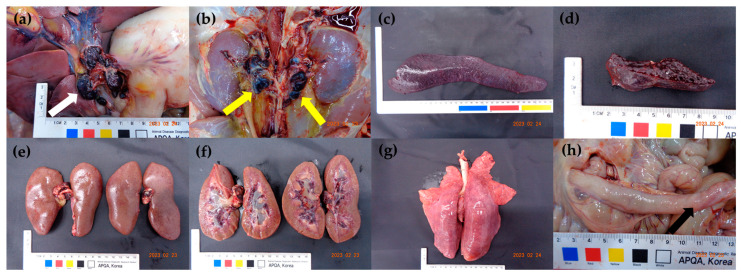
Gross lesions at necropsy. (**a**) Enlargement and hemorrhage of the gastrohepatic lymph nodes in pig no. 3 (white arrow), (**b**) enlargement and hemorrhage of the renal lymph nodes in pig no. 2 (yellow arrows), (**c**) diffusely enlarged dark black spleen, (**d**) congested and friable spleen in pig no. 5, (**e**) numerous petechiae on the cortical surfaces in pig no. 8, (**f**) hemorrhage in the renal medulla and pelvis in pig no. 8, (**g**) non collapsed lungs with interlobular septa edema in pig no. 1, and (**h**) petechiae on the ileal serosa in pig no. 2 (black arrow).

The gross lesions observed in all the pigs included hemorrhagic enlargement in the lymph nodes and splenomegaly with infarction. Renal lesions, including petechiae on the surface of the kidneys and hemorrhage in the renal pelvis, were observed in 8 of 10 pigs except for pigs no. 9 and 10. The pathological lesions observed in some pigs included interstitial pneumonia (7/10), petechiae on the ileal serosa (7/10), and tonsillar erythema (7/10). Only pig no. 8, which had lameness owing to severe joint swelling in the hind limb, showed hemorrhage in the thigh muscles. The gross lesions in each pig are summarized in Table 3. The major gross lesions observed in the challenged pigs are shown in Figure 3.

### 3.4. Histopathological Lesions

**Figure 4:**
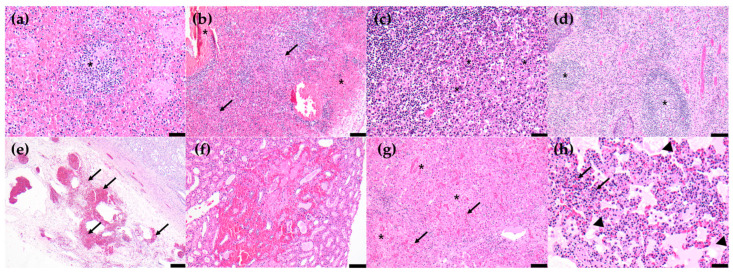
Histopathological lesions. (**a**) Severe lympholysis (asterisk) in the white pulp, spleen, scale bar = 50 µm, (**b**) severe hemorrhage (asterisks) and lympholysis (black arrows) in the gastrohepatic lymph node, scale bar = 100 µm, (**c**) severe lympholysis (asterisks) in the mesenteric lymph node, scale bar = 50 µm, (**d**) severe lympholysis (asterisks) in the tonsils, scale bar = 100 µm, (**e**) severe hemorrhages (black arrows) in the renal pelvis, scale bar = 200 µm, (**f**) renal cortical hemorrhages, scale bar = 100 µm, (**g**) multifocal hepatocellular necrosis (asterisks) with hemorrhages (black arrows), scale bar = 100 µm, and (**h**) infiltration of necrotized inflammatory cells (black arrows) in the alveolar lumen with proteinaceous fluid (arrow heads), lungs, scale = 50 µm.

The major histopathological lesions of the pigs in the experiment were moderate-to-severe lymphoid and histiocytic cell lysis with hemorrhages in lymphoid organs, including the spleen, five lymph nodes, and the tonsils. Mild and moderate multifocal hemorrhages on the renal cortex and medulla with multifocal tubular degeneration and mild-to-moderate hepatocellular necrosis with periportal mononuclear cell infiltration were also observed in all pigs. In some pigs (6/10), mild interstitial pneumonia with interstitial edema was observed. The spleen showed the highest histopathological scores in both groups, followed by the lymph nodes, tonsils, kidneys, liver, and lungs. Histological differences between the two groups were not found. The histopathological lesions and scores are shown in Table 4 and Figure 4, respectively.

## 4. Discussion

According to the criteria for the clinical course and pathogenicity of ASFV [2], the clinical signs, survival period, mortality rate, and pathological lesions observed in animals challenged with the two Korean ASFV isolates corresponded to those of the acute form of illness. This clinical disease progression of both Korean strains is similar to that of the highly virulent p72 genotype II strains, including Georgia 2007/1, Armenia 07, Pig/Heilongjiang/2018, VNUA/HY/Vietnam, and ASFV Mongolia/2019 [25,26,27,28,29]. The results demonstrated that both Korean ASFV strains were highly virulent. There were no changes in pathogenicity compared with those of four Korean ASFV strains isolated from pig farms from 2019 to 2021 [20].

The common clinical signs in all the pigs inoculated with the two Korean ASFV strains were pyrexia, anorexia, depression, recumbency, and death at 8–10 dpi. Respiratory difficulties, joint swelling, ocular discharge, diarrhea, melena, and epistaxis were also observed in some pigs. The pathological lesions observed at necropsy in all the challenged pigs included enlarged hemorrhagic lymph nodes and splenomegaly with infarction. In a previous study with four Korean strains isolated in 2019–2021, some pigs died suddenly at 4–5 dpi without any clinical presentation. The observed clinical manifestations varied slightly from those observed in the animal experiment. Infarcted splenomegaly was observed in all the pigs in this study; however, splenic lesions were not typical in the previous study with Korean ASFV strains [20]. Clinical manifestations varied based on the individual pig, resulting from virulence and host-virus interactions. These data can be used to educate farmers, veterinary officers, and clinical veterinarians.

The disease dynamics of the two Korean isolates were very similar. Viremia was detected at 3–4 dpi, followed by virus shedding via the oral, nasal, and rectal routes and the observation of clinical signs. The viral genome continued to be detected from their first detection to death in the samples obtained from all the challenged pigs, as described previously [25,30,31,32]. However, the viral loads varied. EDTA-whole blood had the highest viral load compared to the swab samples. Although the viral load in the nasal and rectal swabs obtained from the pigs with epistaxis and melena increased, it did not exceed that in the whole blood. Individual swab samples could be considered an alternative; however, owing to the delay of 1–2 days in the detection time compared to whole blood samples, infected pigs in the incubation period may not be detected. In light of the veterinary surveillance system relying on periodic sampling and diagnosis, the risk of misdiagnosis can increase, thereby leading to a more extensive epidemic. Therefore, whole blood is the optimal and reliable sample in the regions where highly virulent ASFV strains are circulating, as described previously [33]. In dead pigs, the ASFV genome was detected in all the seven organs collected at necropsy. The viral loads in the spleen, liver, and lymph nodes were higher than those in other organs. Tissue distribution can differ depending on the virus strain, infection dose and route, and virus-host interactions.

The most characteristic *post-mortem* lesion of acute ASF is hemorrhagic splenomegaly [34,35], while the second most prominent lesion is multifocal hemorrhagic lymphadenitis, which can appear as a marble shape. The most affected lymph nodes are the gastrohepatic and renal lymph nodes [36,37,38]. As the main target cells for ASFV are monocytes and macrophages [34,39], lymphoid organs and tissues, such as the spleen, lymph nodes, thymus, and tonsils, where a large proportion of B and T lymphocytes and macrophages undergoing apoptosis and necrosis during acute ASFV infection [40,41], are the most affected. Histopathological characteristics were clearly identified in pigs experimentally infected with Korean ASFV strains. This tendency was highly similar in other histopathological studies with different highly virulent ASFV strains [26,42,43].

## 5. Conclusions

Since the ASF outbreak in Georgia in 2007, the ASF status worldwide has drastically changed. In particular, the ASF situation in Asia is challenging; to date, virulent genotype II ASFV strains are dominant in this region. Various attenuated ASFV strains emerged between 2020 and 2021 in China [18,19]. Furthermore, genotype I and II recombinant ASFV strains with high virulence, which were not protected by a live attenuated vaccine derived from genotype II virus, were detected in some provinces of China [44]. Several commercial attenuated vaccines against ASFV have recently been used in on-farm trials in Vietnam. In addition to this worsening situation in neighboring countries, South Korea should cope with ASFV circulation control in wild boars, which can result in the natural attenuation of circulating viruses. Although concerns about the emergence of new ASFV strains with low pathogenicity in the country have been increasing, these concerns are likely to be just alarmism until January 2023. In response to the introduction and emergence of attenuated viruses, efforts to monitor the changes in pathogenicity of ASFV strains circulating in the country should be continuously made to maintain effective quarantine measures.

## Figures and Tables

**Table 1:** Information on the ASF outbreaks at pig farms in South Korea from 2022 to January 2023.

Date of the Outbreak		Province	City/County	Number of Pigs (Production Type)	Classification	Clinical Signs	Presence of Hemorrhagic Gross Lesions at Necropsy
1	26 May 2022	Gangwon	Hongcheon	1175 (farrow-to-finish)	Notification	Death	Yes
2	18 August 2022	Gangwon	Yanggu	5614 (farrow-to-finish)	Notification	Death	Yes
3	18 September 2022	Gangwon	Chuncheon	8243 (farrow-to-finish)	Notification	Anorexia, fever, abortion, death	Yes
4	19 September 2022	Gangwon	Chuncheon	6824 (farrow-to-finish)	Active surveillance (Quarantine zone)	None	No necropsy
5	28 September 2022	Gyeonggi	Gimpo	5203 (farrow-to-finish)	Notification	Anorexia, abortion	No necropsy
6	28 September 2022	Gyeonggi	Paju	1133 (farrow-to-finish)	Notification	Anorexia, abortion, death	Yes
7	9 November 2022	Gangwon	Cheorwon	5499 (farrow-to-finish)	Notification	Anorexia, death	Yes
8	5 January 2023	Gyeonggi	Pocheon	8444 (breeding)	Active surveillance (Slaughterhouse)	None	No
9	11 January 2023	Gangwon	Cheorwon	1976 (fattening)	Active surveillance	None	No necropsy
10	22 January 2023	Gyeonggi	Gimpo	2009 (farrow-to-finish)	Notification	Anorexia, fever, death	Yes

**Table 2:** Clinical characteristics and viremia and virus shedding by oral, nasal, and rectal routes of pigs infected with two ASFV isolates from domestic pig farms in South Korea from 2022 to January 2023. All the data are shown as average ± standard deviation.

Group (Number of Pigs)	Inoculated ASFV Strain (Date of Outbreak)	Total Survival Days	Days to Onset of Fever	Clinical Signs		Viremia		Virus Shedding					
								Oral Route		Nasal Route		Rectal Route	
				Days to Onset	Max Score	Days to Onset	Max Titer ^1^	Days to Onset	Max Titer ^1^	Days to Onset	Max Titer ^1^	Days to Onset	Max Titer ^1^
1 (*n* = 5)	Korea/Pig/Hongcheon/2022 (26 May 2022)	9.8 ± 0.4	5.4 ± 0.5	6.6 ± 0.9	14.4 ± 7.6	3.2 ± 0.4	6.9 ± 0.2	4.6 ± 0.5	5.1 ± 0.3	5.2 ± 0.4	6.0 ± 0.2	4.8 ± 0.4	5.4 ± 0.4
2 (*n* = 5)	Korea/Pig/Pocheon/2023 (5 January 2023)	8.8 ± 0.8	5.6 ± 0.9	7.0 ± 0	11.8 ± 3.2	3.2 ± 0.4	7.1 ± 0.2	4.8 ± 0.8	3.2 ± 0.7	4.4 ± 0.5	4.8 ± 0.8	4.4 ± 0.5	4.4 ± 0.7

^1^ log_10_ genome copies/µL.

**Table 3:** Summary of the clinical signs and gross lesions in pigs inoculated with two Korean ASFV strains.

Clinical Signs and Gross Lesions			Group 1					Group 2					Total Frequency	
			1	2	3	4	5	6	7	8	9	10		
Clinical signs	Total days survival		10	9	10	10	10	10	9	9	8	8	-	
	Fever (>40.0 °C)		+	+	+	+	+	+	+	+	+	+	10/10	(100.0%)
	Anorexia		+	+	+	+	+	+	+	+	+	+	10/10	(100.0%)
	Depression		+	+	+	+	+	+	+	+	+	+	10/10	(100.0%)
	Recumbency		+	+	+	+	+	+	+	+	+	+	10/10	(100.0%)
	Labored breathing and/or cough		+	+	+	+	+	–	+	+	–	+	7/10	(70.0%)
	Joint swelling		+	–	+	–	+	+	+	+	–	–	6/10	(60.0%)
	Ocular discharge		–	+	+	+	+	–	–	–	–	–	4/10	(40.0%)
	Diarrhea		–	–	–	+	+	–	–	–	–	+	3/10	(30.0%)
	Melena		–	–	+	–	+	–	+	–	–	–	3/10	(30.0%)
	Epistaxis		–	–	+	–	–	–	–	–	–	–	1/10	(10.0%)
	Skin hemorrhage		–	–	–	–	–	–	–	–	–	–	0/10	(0%)
	Hematuria		–	–	–	–	–	–	–	–	–	–	0/10	(0%)
	Vomit		–	–	–	–	–	–	–	–	–	–	0/10	(0%)
Gross lesions	Lymph nodes	Enlargement	+	+	+	+	+	+	+	+	+	+	10/10	(100.0%)
		Hemorrhage	+	+	+	+	+	+	+	+	+	+	10/10	(100.0%)
	Spleen	Enlargement	+	+	+	+	+	+	+	+	+	+	10/10	(100.0%)
		Infarction	+	+	+	+	+	+	+	+	+	+	10/10	(100.0%)
	Kidneys	Petechiae	+	+	+	+	+	+	+	+	–	–	8/10	(80.0%)
		Hemorrhage in pelvis	+	+	+	+	+	+	+	+	–	–	8/10	(80.0%)
	Lung	Interstitial pneumonia	+	+	–	+	+	+	+	–	+	–	7/10	(70.0%)
	Intestine	Petechiae on ileal serosa	+	+	–	+	+	+	–	+	+	–	7/10	(70.0%)
	Tonsils	Erythema	+	+	–	+	–	+	+	+	+	–	7/10	(70.0%)
	Hindlimb	Hemorrhage	–	–	–	–	–	–	–	+	–	–	1/10	(10.0%)

**Table 4:** Histopathological scores in the tissues of the two groups shown as average ± standard deviation.

Group	Spleen	Lymph Nodes					Tonsils	Kidneys	Liver	Lung
		Submandibular	Tracheobronchial	Gastrohepatic	Renal	Mesenteric				
1	3.00 ± 0	2.80 ± 0.45	2.60 ± 0.55	2.80 ± 0.45	3.00 ± 0	2.60 ± 0.55	2.80 ± 0.45	1.40 ± 0.55	1.20 ± 0.45	0.75 ± 0.50
2	3.00 ± 0	2.40 ± 0.89	3.00 ± 0	2.80 ± 0.45	3.00 ± 0	2.80 ± 0.45	2.40 ± 0.89	1.40 ± 0.55	1.40 ± 0.55	0.60 ± 0.55

## Data Availability

The data used in this study are available upon request from the corresponding author.
